# Alzheimer Disease in Breast Cancer Survivors

**DOI:** 10.1001/jamanetworkopen.2025.16468

**Published:** 2025-06-20

**Authors:** Su-Min Jeong, Wonyoung Jung, Hyeonjin Cho, Hea Lim Choi, Keun Hye Jeon, Ki-Woong Nam, Yun-Gyoo Lee, Bongseong Kim, Kyungdo Han, Dong Wook Shin

**Affiliations:** 1 Department of Medicine, Seoul National University College of Medicine, Seoul, Republic of Korea; 2 Division of Cardiology, Department of Medicine, Perelman School of Medicine, University of Pennsylvania, Philadelphia; 3 Department of Clinical Research Design and Evaluation, The Samsung Advanced Institute for Health Sciences & Technology (SAIHST), Sungkyunkwan University, Seoul, Republic of Korea; 4 Department of Family Medicine, Executive Healthcare Clinic, Severance Hospital, Yonsei University College of Medicine, Seoul, Republic of Korea; 5 Department of Family Medicine, CHA Gumi Medical Center, CHA University School of Medicine, Gumi, Republic of Korea; 6 Department of Neurology, Seoul Metropolitan Government-Seoul National University Boramae Medical Center, Seoul, Republic of Korea; 7 Division of Hematology & Medical Oncology, Department of Internal Medicine, Samsung Kangbuk Hospital, Sungkyunkwan University School of Medicine, Seoul, Republic of Korea; 8 Department of Medical Statistics, The Catholic University of Korea, Seoul, Republic of Korea; 9 Department of Statistics and Actuarial Science, Soongsil University, Seoul, Republic of Korea; 10 Department of Family Medicine, Supportive Care Center, Samsung Medical Center, Sungkyunkwan University School of Medicine, Seoul, Republic of Korea

## Abstract

**Importance:**

Cancer-related cognitive impairment is a common concern after breast cancer treatment. However, the association between breast cancer survivorship and the risk of Alzheimer dementia (AD) remains unclear.

**Objective:**

To evaluate the risk of AD among breast cancer survivors compared with cancer-free controls and examine the potential association of cancer treatments with AD risk.

**Design, Setting, and Participants:**

This retrospective cohort study used data from the Korean National Health Insurance Service. A total of 70 701 patients who underwent breast cancer surgery between January 1, 2010, and December 31, 2016, were included and matched with cancer-free controls (1:3). Participants were followed up for a median (IQR) of 7.3 (5.7-9.0) years. Data analysis was performed from January 2024 to June 2024.

**Exposures:**

Breast cancer diagnosis, surgery, and subsequent treatments, including anthracycline chemotherapy and radiation therapy.

**Main Outcomes and Measures:**

The primary outcome was the incidence of AD. Subdistribution hazard ratios (SHRs) and 95% CIs were calculated using competing risk regression models, adjusting for sociodemographic factors and comorbidities.

**Results:**

Among 70 701 breast cancer survivors (mean [SD] age, 53.1 [8.5] years), 1229 cases of AD were detected, with an incidence rate of 2.45 per 1000 person-years. Survivors exhibited a slightly lower risk of AD compared with cancer-free controls (SHR, 0.92; 95% CI, 0.86-0.98), especially among individuals 65 years or older (SHR, 0.92; 95% CI, 0.85-0.99). However, landmark analyses found that this lower risk did not persist beyond 5 years of survival. Cancer treatment with radiation therapy (adjusted HR, 0.77; 95% CI, 0.68-0.87) was associated with reduced risk of AD among survivors.

**Conclusions and Relevance:**

This cohort study of breast cancer survivors found a lower risk of AD compared with cancer-free controls, despite common concerns about cognitive decline after treatment. The findings suggest certain cancer treatments potentially have benefits for lower AD risk. Further research is needed to assess the long-term risk of AD in this population.

## Key Points

**Question:**

Is breast cancer survivorship associated with the risk of Alzheimer dementia (AD), and how are cancer treatments associated with this risk?

**Findings:**

In this cohort study of 70 701 breast cancer survivors matched 1:3 with cancer-free controls, breast cancer survivors demonstrated a lower risk of AD compared with controls, particularly among those 65 years or older, although the lower risk did not persist beyond 5 years. Treatment with radiation therapy was associated with reduced AD risk.

**Meaning:**

Breast cancer survivors may have a slightly lower risk of AD compared with cancer-free individuals, potentially influenced by cancer treatments, underscoring the need for further research on long-term neurocognitive outcomes in this population.

## Teaser

This cohort study investigates the risk of Alzheimer disease (AD) among breast cancer survivors compared with matched controls without a history of cancer and explores cancer treatment factors potentially associated with AD.

## Introduction

Breast cancer is the most common cancer among women worldwide, with more than 2.3 million new cases in 2022, accounting for 11.6% of all cancer cases globally.^1^ Survival rates for breast cancer have substantially improved, exceeding 93% for early-stage disease due to early detection and advances in treatment.^2^ This remarkable increase in survival has shifted the focus toward long-term health consequences and quality of life for breast cancer survivors, including cognitive function and risk of dementia.

A substantial number of breast cancer survivors report cancer-related cognitive impairment, experiencing difficulties in concentration and memory during and after cancer treatment.^3^ However, evidence regarding the risk of Alzheimer dementia (AD) among breast cancer survivors remains mixed and inconclusive and may vary by age at diagnosis, treatment received, and time since treatment (eTable 1 in Supplement 1). Early studies from Northern Italy and Denmark on the standardized incidence rate (SIR) of AD in patients with cancer reported a low incidence of AD in patients with cancer overall (SIR, 0.68; 95% CI, 0.34-1.00) and in breast cancer survivors specifically (SIR, 0.95; 95% CI, 0.91-1.00).^4,5^ In contrast, a Swedish study comparing 26 741 five-year breast cancer survivors and a cancer-free group found a 35% increased risk of AD among those who were diagnosed with cancer at an age older than 65 years (subdistribution hazard ratio [SHR], 1.35; 95% CI, 1.05-1.75) compared with those without cancer. This increased risk was not present in younger survivors (SHR, 1.04; 95% CI, 0.83-1.30), suggesting different associations by age at cancer diagnosis.^6^ In Asia, breast cancer is more commonly diagnosed in the premenopause period. A Taiwanese nationwide study reported no increase in the risk of AD compared with that of a cancer-free group (adjusted HR [AHR], 0.95; 95% CI, 0.86-1.04), and individuals receiving tamoxifen had a lower risk of dementia (AHR, 0.83; 95% CI, 0.69-0.98).^7^ A recent Korean population-based cohort study involving 15 407 breast cancer survivors older than 50 years revealed a marked decreased risk of dementia (of which 88% were classified as having AD) (HR, 0.091; 95% CI, 0.075-0.111) compared with that of control groups who had cataracts, and there was no risk difference associated with chemotherapy or endocrine therapy.^8^

Previous studies are limited by several methodologic issues. To our knowledge, no study has considered important risk factors, such as smoking, alcohol, physical activity, and body mass index.^4,5,6,7,8^ Only one study accounted for competing risk of death despite higher mortality compared with the general population.^6^ A Korean cohort study used matched controls with a history of cataracts,^8^ a condition that in itself might increase the risk of dementia, affecting the much lower risk of dementia observed among breast cancer survivors.^9,10^ In addition, the small study population and number of AD cases (approximately <1500 AD cases) in other studies might have hindered stratification analyses by treatment modality and various confounding factors.^6,8^ Therefore, we aimed to investigate the risk of AD among breast cancer survivors compared with matched controls without a history of cancer, exploring whether there was an association with cancer treatment using the Korean National Health Insurance Service (K-NHIS) database.

## Methods

### Data Source and Study Setting

The K-NHIS provides universal health insurance for 97% of the Korean population. This national health insurance is mandatory for all Korean citizens and medical facilities, and all medical facilities are reimbursed by the K-NHIS. The K-NHIS database includes information on sociodemographic variables (insurance premium and residential area), all medical claims that include diagnostic codes specified in the *International Statistical Classification of Diseases and Related Health Problems, Tenth Revision (ICD-10), *prescriptions, and procedures. In addition, the K-NHIS offers biennial national health screening programs for those aged 20 years or older to assess individual health status based on questionnaires on medical history and lifestyle behaviors (smoking, alcohol consumption, and physical activity), anthropometric measurements, and laboratory tests.^11^ This nationwide dataset has been widely used in various epidemiologic studies regarding dementia.^12,13,14^ This study was approved by the institutional review board of Samsung Medical Center. Due to the anonymized nature of the data, the requirement for informed consent from participants was waived by the institutional review board. This study adhered to the Strengthening the Reporting of Observational Studies in Epidemiology (STROBE) reporting guideline for cohort studies.

### Study Population

Initially, 126 566 patients with breast cancer who had undergone related surgery within 1 year from breast cancer diagnosis between January 1, 2010, and December 31, 2016, were included. Among this population, we excluded those diagnosed with any other cancers before their breast cancer diagnosis (n = 5514; prior cancer diagnosis between January 1, 2002, and the date when breast cancer was diagnosed), those younger than 40 years (n = 13 396), those diagnosed with all-cause dementia between January 1, 2002, and cancer diagnosis (n = 690), and those with missing values (n = 2387). Ultimately, 100 905 patients with breast cancer were included. The control group was selected from among Korean women with no history of cancer, as determined from claims data between 2002 and 2016. Controls were matched to breast cancer survivors by age (1:3; n = 302 712), and the index date for the control group was assigned based on the diagnosis date of matched breast cancer survivors. Then individuals who underwent a health examination within 2 years prior to breast cancer diagnosis (n = 70 701) and their controls (n = 180 360) were included in final analysis to obtain information for covariates (Figure).

**Figure.**
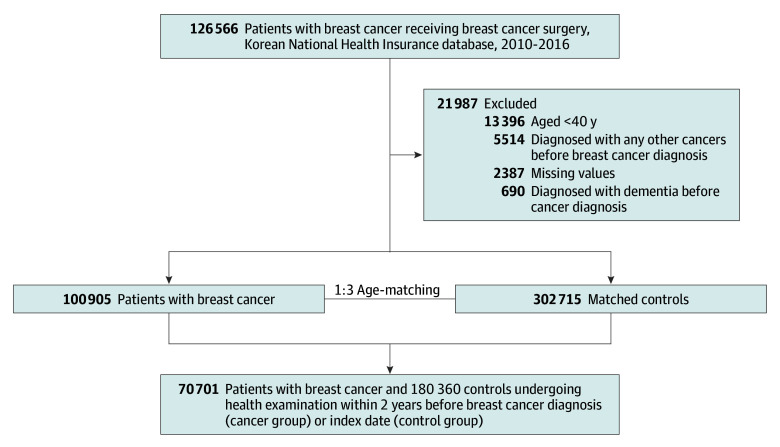
Flowchart of the Study Population

### Identification of Breast Cancer Survivors

Breast cancer survivors were primarily identified based on the presence of *ICD-10* code C50. In addition, a specialized claim code (V193) from the rare and intractable disease (RID) registration program was considered. Since 2005, the K-NHIS has used an RID registration program that reduces patient medical expenses to only 5% of the total costs related to cancer treatment. The specific code is assigned to patients only after a definitive diagnosis of breast cancer by a health professional. The accuracy of cancer diagnoses based on the RID registration program has been validated with sufficiently high sensitivity for breast cancer (98.1%).^15^

### Study Outcomes and Follow-Up

The primary outcome of this study was the incidence of newly diagnosed AD. Newly diagnosed AD was defined based on at least 1 prescription for antidementia medications (donepezil, rivastigmine, galantamine, or memantine) under the relevant *ICD-10* codes (F00 and G30) during the follow-up period.^16^ To be prescribed an antidementia medication under the K-NHIS, patients’ cognitive function needs to meet the following criteria: (1) Mini-Mental State Examination score of 26 or less and (2) either a Clinical Dementia Rating of 1 or greater or a Global Deterioration Scale score of 3 or greater.^17^ The participants were followed up until the date of AD diagnosis, the end of the follow-up period, December 31, 2020, or death, whichever came first.

### Covariates

The participants in the lowest 25% of insurance premiums (a proxy of income status under the Korean Social Health Insurance System) were categorized as the lower-income group. Based on a self-reported questionnaire regarding health behaviors, smoking status (never or ex-smoker vs current smokers), alcohol consumption (nondrinkers vs drinkers), and physical activity status were classified. Alcohol consumption was assessed using the question, “How frequently have you consumed alcohol in the past year?” Participants were classified as nondrinkers if they answered, “No, I do not drink.” Physical activity status was evaluated based on the frequency and duration of exercise per session. Regular physical activity was defined as engaging in vigorous-intensity exercise for at least 20 minutes on 3 or more days per week or moderate-intensity exercise for at least 30 minutes on 5 or more days per week using a modified version of the International Physical Activity Questionnaire.^18^ Obesity was defined as body mass index of 25 or more (calculated as weight in kilograms divided by height in meters squared) using the Asian-Pacific criteria.

Comorbidities were assessed 1 year prior to the index date. The presence of comorbidities was determined by the combination of prescription(s), relevant diagnostic codes, and clinical measurements or laboratory tests as follows: diabetes (*ICD-10* codes E11-E14 with antidiabetic medications or fasting glucose levels ≥126 mg/dL [to convert to millimoles per liter, multiply by 0.0555]), hypertension (*ICD-10* codes I10-I13 and I15 with antihypertensive medications or blood pressure ≥140/90 mm Hg), dyslipidemia (*ICD-10* code E78 with lipid-lowering medications or total cholesterol levels ≥240 mg/dL [to convert to millimoles per liter, multiply by 0.0259]), and chronic kidney disease (glomerular filtration rate <60 mL/min/1.73 m^2^ as estimated by the Modification of Diet in Renal Disease formula).

Information on breast cancer treatment was obtained from claims data within 1 year of diagnosis. Clinical guidelines recommend initiating adjuvant chemotherapy within 120 days of diagnosis,^19^ and in Korea, quality assessment indicators for cancer care include the initiation of adjuvant therapy within 8 weeks after surgery.^20^ Treatment with chemotherapy was defined by at least 1 cycle of chemotherapeutic agents (anthracycline, cyclophosphamide, fluorouracil, taxane, methotrexate, and cisplatin). Endocrine therapy included the use of tamoxifen and aromatase inhibitors (anastrozole, exemestane, and letrozole) as the initial prescription if there was switching to another hormonal therapy.^21^

### Statistical Analysis

The baseline characteristics are depicted as mean (SD) and number (percentage) for continuous and categorical variables, respectively. The significance of differences in means and proportions was assessed by 2-tailed *t* tests and χ^2^ tests.

To estimate the risk of AD incidence considering death as a competing event, the Fine-Gray subdistribution hazard model was used to obtain SHRs and 95% CIs.^22^ The proportional hazards assumption was evaluated using the Schoenfeld residuals, and no significant violation was found. Subdistribution hazards across the groups were compared by using the Gray test (eFigure 1 in Supplement 1). In addition to a crude model (model 1), model 2 was adjusted for age, income levels, and residential location, and model 3 was additionally adjusted for body mass index, comorbidities (diabetes, hypertension, dyslipidemia, and chronic kidney disease) and health-related behaviors (smoking, alcohol consumption, and physical activity). Stratification analysis by age group (≤50, 51-64, and ≥65 years) was conducted to evaluate the different association(s) by age.

Landmark analyses, which included individuals who were event free at each of 3 time points (1, 3, and 5 years after breast cancer diagnosis), were conducted as a sensitivity analysis to estimate the risk of AD. This analysis allowed us to demonstrate the risk of AD according to survivor-years since cancer diagnosis. Among the breast cancer survivors, the risk factors for AD incidence were explored.

Statistical analyses were performed using SAS software, version 9.4 (SAS Institute Inc). A 2-sided *P* < .05 was considered statistically significant. Data analysis was performed from January 2024 to June 2024.

## Results

### Baseline Characteristics

Of the 70 701 breast cancer survivors (mean [SD] age, 53.1 [8.5] years), 1229 cases of AD were detected, with an incidence rate of 2.45 per 1000 person-years. The baseline characteristics of the study population are presented in Table 1. Breast cancer survivors tended to have more comorbidities, such as diabetes (4704 [6.7%] vs 11 571 [6.4%]), hypertension (15 633 [22.1%] vs 39 061 [21.7%]), and dyslipidemia (12 770 [18.1%] vs 31 463 [17.4%]), than did controls without cancer. In terms of cancer treatment, 50 681 breast cancer survivors (71.7%) underwent radiotherapy. Cyclophosphamide (40 144 [56.8%]) and anthracycline (35 441 [50.1%]) were the most commonly used chemotherapeutic agents (eTable 2 in Supplement 1). Of the breast cancer survivors, 33 232 (47.0%) and 21 240 (30.0%) were treated with tamoxifen and aromatase inhibitors, respectively.

**Table 1.** Baseline Characteristics of the Study Participants

Characteristic	No. (%)^a^		*P* value
	Control group (n = 180 360)	Breast cancer group (n = 70 701)	
Age, mean (SD), y	53.3 (8.5)	53.1 (8.5)	<.001
Income status of lower 25%	44 180 (24.5)	16 355 (23.1)	<.001
Urban location	83 525 (46.3)	35 223 (49.8)	<.001
Current smoker	6115 (3.4)	2687 (3.8)	<.001
Alcohol use	42 187 (23.4)	16 735 (23.7)	.14
Regular physical activity	34 477 (19.1)	13 298 (18.8)	.08
BMI, mean (SD)	23.7 (3.3)	23.7 (3.3)	.04
BMI ≥25	54 961 (30.5)	21 863 (30.9)	.03
Waist circumference (SD), cm	77.3 (8.6)	77.4 (8.6)	.03
Comorbidity			
Diabetes	11 571 (6.4)	4704 (6.7)	.03
Hypertension	39 061 (21.7)	15 633 (22.1)	.01
Dyslipidemia	31 463 (17.4)	12 770 (18.1)	<.001
Chronic kidney disease	549 (0.3)	244 (0.4)	.10

Abbreviation: BMI, body mass index (calculated as weight in kilograms divided by height in meters squared).

^a^ Unless otherwise indicated.

### AD Risk Among Breast Cancer Survivors vs Matched Controls

During a median (IQR) of 7.3 (5.7-9.0) years of follow-up, 1229 and 3430 AD cases were detected among breast cancer survivors and cancer-free controls, with incidence rates of 2.45 and 2.63 (per 1000 person-years), respectively (Table 2). Breast cancer survivors had a lower risk of AD (SHR, 0.92; 95% CI, 0.86-0.98) than did controls. In stratification analysis, there was no significant interaction by age group; however, a significant association was noted in those aged 65 years or older (SHR, 0.92; 95% CI, 0.85-0.99) (eFigure 2 in Supplement 1).

**Table 2.** Adjusted SHRs for Risk of Alzheimer Disease in Breast Cancer Survivors Compared With the Control Group Without Cancer by Age Category

Group	No. of participants	No. of cases	Duration, person-years	Incidence rate per 1000 person-years	SHR (95% CI)		
					Model 1^a^	Model 2^b^	Model 3^c^
**All ages**							
Control	180 360	3430	1 302 576.2	2.63	1.0 [Reference]	1.0 [Reference]	1.0 [Reference]
Breast cancer	70 701	1229	500 740.4	2.45	0.90 (0.85-0.97)	0.93 (0.87-0.99)	0.92 (0.86-0.98)
**Age ≤50 y**							
Control	77 202	92	562 696.8	0.16	1.0 [Reference]	1.0 [Reference]	1.0 [Reference]
Breast cancer	31 441	33	225 902.1	0.15	0.87 (0.58-1.29)	0.88 (0.59-1.32)	0.88 (0.59-1.31)
**Age 51 to ≤64 y**							
Control	81 707	914	589 079.0	1.55	1.0 [Reference]	1.0 [Reference]	1.0 [Reference]
Breast cancer	31 197	326	219 782.7	1.48	0.93 (0.82-1.05)	0.94 (0.83-1.07)	0.93 (0.82-1.06)
**Age ≥65 y**							
Control	21 451	2424	150 800.4	16.07	1.0 [Reference]	1.0 [Reference]	1.0 [Reference]
Breast cancer	8063	870	55 055.6	15.80	0.94 (0.87-1.02)	0.93 (0.86-1.01)	0.92 (0.85-0.99)
*P* for interaction	NA	NA	NA	NA	.90	.95	.96

Abbreviations: NA, not applicable; SHR, subdistribution hazard ratio.

^a^ Model 1: unadjusted.

^b^ Model 2: adjusted for age, income, and residential location.

^c^ Model 3: adjusted for age, income, residential location, body mass index, diabetes, hypertension, dyslipidemia, chronic kidney disease, smoking, alcohol consumption, and physical activity.

### Treatment Factors and AD Risk Among Breast Cancer Survivors

When AD risk was analyzed by treatment modality, radiation therapy was associated with significantly lower risk (AHR, 0.77; 95% CI, 0.68-0.87). Anthracycline use was not associated with risk of AD (AHR, 0.86; 95% CI, 0.73-1.01). There was no association of treatment or AD with trastuzumab (AHR, 1.00; 95% CI 0.82-1.23), taxane (AHR, 1.08; 95% CI, 0.90-1.30), endocrine therapy (including those treated only with tamoxifen [AHR, 0.92; 95% CI, 0.78-1.09], aromatase inhibitor [AHR, 1.04; 95% CI, 0.90-1.20]), or combined tamoxifen and aromatase inhibitors (AHR, 1.09; 95% CI, 0.73-1.62) (Table 3).

**Table 3.** Factors Associated With Alzheimer Disease Among Breast Cancer Survivors

Risk factor	No. of participants	No. of cases	Duration, person-years	Incidence ratio per 1000 person-years	AHR (95% CI)^a^
Treatment					
Anthracycline					
No	35 260	860	249 237.4	3.45	1.0 [Reference]
Yes	35 441	369	251 503.0	1.47	0.86 (0.73-1.01)
Taxane					
No	51 812	1029	372 044.6	2.77	1.0 [Reference]
Yes	18 889	200	128 695.8	1.55	1.08 (0.90-1.30)
Trastuzumab					
No	60 460	1102	430 113.8	2.56	1.0 [Reference]
Yes	10 241	127	70 626.6	1.80	1.00 (0.82-1.23)
Endocrine therapy					
No	17 927	346	124 723.7	2.77	1.0 [Reference]
Tamoxifen	31 534	277	228 755.1	1.21	0.92 (0.78-1.09)
Aromatase inhibitor	19 542	570	136 585.0	4.17	1.04 (0.90-1.20)
Both	1698	36	10 676.6	3.37	1.09 (0.73-1.62)
Radiation therapy					
No	20 020	599	140 387.4	4.27	1 [Reference]
Yes	50 681	630	360 353.0	1.75	0.77 (0.68-0.87)
Other covariates					
Residential location					
Urban	35 223	580	250 454.6	2.32	1.0 [Reference]
Rural	35 478	649	250 285.8	2.59	1.10 (0.98-1.24)
Income					
High	54 346	948	386 889.5	2.45	1.0 [Reference]
Lower 25%	16 355	281	113 850.9	2.47	1.24 (1.08-1.43)
BMI					
<25	48 838	657	347 307.9	1.89	1.0 [Reference]
≥25	21 863	572	153 432.5	3.73	1.16 (0.96-1.40)
Smoking status					
Never or ex-smoker	68 014	1177	481 928.9	2.44	1.0 [Reference]
Current smoker	2687	52	18 811.5	2.76	2.04 (1.53-2.72)
Alcohol consumption					
None	53 966	1121	383 594.0	2.92	1.0 [Reference]
> 0 g/d	16 735	108	117 146.4	0.92	0.89 (0.72-1.10)
Physical activity					
None	57 403	1016	406 894.2	2.50	1.0 [Reference]
Regular exercise	13 298	213	93 846.2	2.27	0.95 (0.81-1.10)
Diabetes					
No	65 997	927	468 615.3	1.98	1.0 [Reference]
Yes	4704	302	32 125.1	9.40	1.58 (1.36-1.82)
Hypertension					
No	55 068	541	391 198.9	1.38	1.0 [Reference]
Yes	15 633	688	109 541.5	6.28	1.07 (0.93-1.22)
Dyslipidemia					
No	57 931	733	413 258.0	1.77	1.0 [Reference]
Yes	12 770	496	87 482.4	5.67	1.13 (0.99-1.28)
Chronic kidney disease					
No	70 457	1209	499 199.5	2.42	1.0 [Reference]
Yes	244	20	1540.9	12.98	3.11 (1.98-4.88)

Abbreviations: AHR, adjusted hazard ratio; BMI, body mass index (calculated as weight in kilograms divided by height in meters squared).

^a^ Adjusted for age, income, residential location, body mass index, diabetes, hypertension, dyslipidemia, chronic kidney disease, smoking, alcohol consumption, and physical activity.

### Modifiable Risk Factors of AD Among Breast Cancer Survivors

Among other covariates, lower income was associated with a higher risk of AD (AHR, 1.24; 95% CI, 1.08-1.43). Compared with never or ex-smokers, current smokers had a higher risk of AD (AHR, 2.04; 95% CI, 1.53-2.72). The comorbidities of diabetes (AHR, 1.58; 95% CI, 1.36-1.82) and chronic kidney disease (AHR, 3.11; 95% CI, 1.98-4.88) were associated with a higher risk of AD (Table 3).

### Landmark Analyses at 6-Month and 1-, 3-, and 5-Year Follow-Ups

Table 4 presents the 6-month, 1-year, 3-year, and 5-year landmark analyses of risk of AD among breast cancer survivors. The results from the 6-month landmark analysis showed that breast cancer survivors had a lower risk of AD compared with controls (HR, 0.92; 95% CI, 0.86-0.99). At the 1-year, 3-year, and 5-year landmark periods, no significant difference in risk of AD was found among breast cancer survivors (1- year landmark: SHR, 0.94; 95% CI, 0.87-1.01; 3-year landmark: SHR, 0.97; 95% CI, 0.90-1.05; 5-year landmark: and SHR, 0.98; 95% CI, 0.89-1.08). As survival time increased, the SHR approached 1.00 (no significant difference in risk for AD compared with controls). These findings were consistent across all age groups (5-years landmark analysis: *P* for interaction = .69).

**Table 4.** Landmark Analyses for Risk of Alzheimer Disease Among Breast Cancer Survivors at 6 Months and 1, 3, and 5 Years

Group	No. of participants	No. of cases	Duration, person-years	Incidence ratio per 1000 person-years	SHR (95% CI)		
					Model 1^a^	Model 2^b^	Model 3^c^
**6-mo Landmark**							
All ages							
Control	180 265	3335	1 212 419.0	2.75	1.0 [Reference]	1.0 [Reference]	1.0 [Reference]
Breast cancer	70 576	1203	465 414.8	2.58	0.91 (0.85-0.97)	0.94 (0.87-1.00)	0.92 (0.86-0.99)
Age ≤50 y							
Control	77 201	91	524 096.1	0.17	1.0 [Reference]	1.0 [Reference]	1.0 [Reference]
Breast cancer	31 407	32	210 187.8	0.15	0.85 (0.57-1.28)	0.87 (0.58-1.298)	0.87 (0.58-1.30)
Age 51-≤64 y							
Control	81 682	889	548 231.7	1.62	1.0 [Reference]	1.0 [Reference]	1.0 [Reference]
Breast cancer	31 150	322	204 193.1	1.58	0.94 (0.83-1.07)	0.96 (0.84-1.09)	0.95 (0.84-1.08)
Age ≥65 y							
Control	21 382	2355	140 091.2	16.81	1.0 [Reference]	1.0 [Reference]	1.0 [Reference]
Breast cancer	8019	849	51 033.9	16.64	0.95 (0.88-1.03)	0.94 (0.87-1.02)	0.93 (0.86-1.01)
*P* for interaction	NA	NA	NA	NA	.88	.89	.90
**1-y Landmark**							
All ages							
Control	180 146	3226	1 122 314.2	2.87	1.0 [Reference]	1.0 [Reference]	1.0 [Reference]
Breast cancer	70 392	1170	430 169.1	2.72	0.92 (0.86-0.98)	0.95 (0.89-1.02)	0.94 (0.87-1.01)
Age ≤50 y							
Control	77 198	90	485 496.1	0.19	1.0 [Reference]	1.0 [Reference]	1.0 [Reference]
Breast cancer	31 348	32	194 495.5	0.16	0.86 (0.58-1.29)	0.88 (0.59-1.32)	0.88 (0.59-1.31)
Age 51-≤64 y							
Control	81 656	865	507 396.7	1.70	1.0 [Reference]	1.0 [Reference]	1.0 [Reference]
Breast cancer	31 087	314	188 632.2	1.66	0.95 (0.83-1.08)	0.97 (0.85-1.10)	0.96 (0.84-1.09)
Age ≥65 y							
Control	21 292	2271	129 421.5	17.55	1.0 [Reference]	1.0 [Reference]	1.0 [Reference]
Breast cancer	7957	824	47 041.4	17.52	0.96 (0.89-1.04)	0.95 (0.88-1.03)	0.94 (0.87-1.02)
*P* for interaction	NA	NA	NA	NA	.88	.91	.92
**3-y Landmark**							
All ages							
Control	179 357	2574	762 725.0	3.37	1.0 [Reference]	1.0 [Reference]	1.0 [Reference]
Breast cancer	68 882	925	290 788.5	3.18	0.93 (0.86-0.99)	0.98 (0.91-1.06)	0.97 (0.90-1.05)
Age ≤50 y							
Control	77 148	63	331 138.1	0.19	1.0 [Reference]	1.0 [Reference]	1.0 [Reference]
Breast cancer	30 882	28	132 257.8	0.21	1.09 (0.70-1.70)	1.11 (0.71-1.74)	1.11 (0.71-1.73)
Age 51-≤64 y							
Control	81 430	698	344 279.4	2.03	1.0 [Reference]	1.0 [Reference]	1.0 [Reference]
Breast cancer	30 409	234	127 062.5	1.84	0.89 (0.77-1.04)	0.91 (0.79-1.06)	0.91 (0.78-1.05)
Age ≥65 y							
Control	20 779	1813	87 307.5	20.77	1.0 [Reference]	1.0 [Reference]	1.0 [Reference]
Breast cancer	7591	663	31 468.2	21.07	0.99 (0.91-1.09)	1.00 (0.92-1.10)	0.99 (0.91-1.09)
*P* for interaction	NA	NA	NA	NA	.42	.48	.47
**5-y Landmark**							
All ages							
Control	157 045	1745	413 162.8	4.22	1.0 [Reference]	1.0 [Reference]	1.0 [Reference]
Breast	59 563	622	157 427.2	3.95	0.93 (0.85-1.02)	0.99 (0.90-1.09)	0.98 (0.89-1.08)
Age ≤50 y							
Control	68 321	42	180 368.5	0.23	1.0 [Reference]	1.0 [Reference]	1.0 [Reference]
Breast	27 115	16	72 201.0	0.22	0.99 (0.55-1.76)	1.00 (0.56-1.79)	1.00 (0.56-1.79)
Age 51-≤64 y							
Control	70 978	481	185 500.7	2.59	1.0 [Reference]	1.0 [Reference]	1.0 [Reference]
Breast	26 088	161	68 227.2	2.36	0.91 (0.76-1.09)	0.92 (0.78-1.11)	0.92 (0.77-1.10)
Age ≥65 y							
Control	17 746	1222	47 293.6	25.84	1.0 [Reference]	1.0 [Reference]	1.0 [Reference]
Breast	6360	445	16 999.0	26.18	1.01 (0.90-1.12)	1.02 (0.91-1.14)	1.01 (0.91-1.13)
*P* for interaction	NA	NA	NA	NA	.63	.69	.69

Abbreviations: NA, not applicable; SHR, subdistribution hazard ratio.

^a^ Model 1: unadjusted.

^b^ Model 2: adjusted for age, income, and residential location.

^c^ Model 3: adjusted for age, income, residential location, body mass index, diabetes, hypertension, dyslipidemia, chronic kidney disease, smoking, alcohol consumption, and physical activity.

## Discussion

In this nationwide population-based cohort study, we investigated the risk of AD among breast cancer survivors compared with age-matched controls without cancer. Breast cancer survivors had an 8% lower risk of AD than did controls, and this association was particularly notable in survivors older than 65 years (SHR, 0.92; 95% CI, 0.85-0.99). In terms of treatment modalities, radiation therapy was associated with lower risk of AD among breast cancer survivors. In the sensitivity analysis, the lower risk of AD did not persist in older breast cancer survivors because survival time increased from 1 year to 5 years.

We found a slightly lower risk of AD among breast cancer survivors, in line with several previous studies^7,8,23,24^ and a meta-analysis^23^ (relative risk [RR], 0.93; 95% CI, 0.87-0.99). In contrast, a Swedish cohort study^6^ reported increased risk of AD among older breast cancer survivors at 5 years; the authors suggested that this difference may have been due to the failure of other studies to consider competing risk. However, one meta-analysis conducted meta-regressions to quantify the effects of study biases on pooled cancer-AD risk estimates and concluded that the inverse association between cancer and AD cannot be explained by competing risks alone.^25^ In our study, we found a lower risk of AD after adjusting for competing risk.

Treatment for cancer has been suggested as a potential mechanism of lower AD risk in breast cancer. One meta-analysis showed a lower risk of dementia and AD in patients with breast cancer treated with chemotherapy (RR, 0.83; 95% CI, 0.73-0.95) or hormonal therapy (RR, 0.83; 95% CI, 0.74-0.94).^23^ Our study also showed a numerically lower risk of AD with anthracycline use (AHR, 0.86; 95% CI, 0.73-1.01), although this result was not statistically significant. Most studies on AD risk after chemotherapy focused on detrimental changes in brain structures^26^ or cognitive function^27^ rather than AD incidence. Cytotoxic chemotherapy has been recognized as a cause of cognitive decline called *chemobrain* in cancer survivors. Chemobrain refers to cognitive dysfunction, including thinking and memory problems, that occur in patients with cancer during and after chemotherapy.^28^ In contrast to AD, chemotherapy-induced cognitive impairment is subtle, typically remains within normal cognitive function range, and does not affect the retrieval of remote memories.^29^ Chemotherapy-induced cognitive impairment does not always lead to the development of AD due to different mechanistic origins and should be distinguished from AD.^30^ In addition, cancer treatment might have benefits against AD development. In vitro, anthracyclines can inhibit and dissolve tau aggregation.^31^ An in vivo study reported that anthracycline significantly reduced the formation of amyloid deposits, suggesting its beneficial effects through the inhibition of fibril growth and facilitation of amyloid deposit clearance.^32^ Dysfunction of autophagy and reduced autophagic flux are both suggested to trigger AD.^33^ Therefore, autophagy inducers, such as anticancer drugs, might manage AD.^34^ Use of taxane-based chemotherapy may impair attention, concentration, and executive function shortly after treatment.^35^ However, research on long-term dementia risk found no association with the use of taxane.^36^

Endocrine therapy reduces estrogen levels, which could be related to an increased risk of dementia.^37,38^ However, in our study, use of tamoxifen and aromatase inhibitors was not significantly associated with AD. Tamoxifen, a selective estrogen receptor modulator that has tissue-specific effects, is widely prescribed for hormone receptor–positive breast cancer. In contrast to the antiestrogen effects of tamoxifen in breast tissue, tamoxifen exerts estrogenic agonist action, showing neuroprotective function against amyloid-β.^39^ Aromatase inhibitors reduce the peripheral levels of estrogen but increase estrogen levels in specific brain regions.^40^ In addition, upstream precursors of estrogen, such as testosterone and androstenedione, are increased by aromatase inhibitors and may benefit cognitive function.^41^ Previous epidemiologic studies also support the use of tamoxifen and aromatase inhibitors to be associated with a lower risk of AD.^7,42^

We found that radiation therapy was associated with lower risk of AD. The effects of therapeutic radiation for breast cancer on AD development have rarely been investigated. An increased risk of dementia in patients with head and neck cancer treated with radiation therapy was noted.^43^ However, the risk of AD could differ, depending on the dose of radiation and site of exposure. A pilot study^44^ found that patients with AD treated with low-dose whole-brain radiotherapy at 3 Gy experienced a temporary improvement in cognitive function via a neuroprotective effect on microglia. The mean unintended dose to the brain from breast cancer radiotherapy was estimated to be approximately 0.2 Gy using a 50-Gy tumor dose.^45^ A US population–based study also reported therapeutic radiation for cancer to be associated with decreased risk of AD for a short period after treatment.^30^ A recent review study also reported that low-dose radiation therapy might reduce astrogliosis and microgliosis and have anti-inflammatory and neuroprotective effects in animal models.^46^ However, indication bias may influence this association because patients who receive radiation therapy are likely to have undergone breast-conserving surgery. Patients who receive breast-conserving surgery plus radiation therapy are likely to be younger, with fewer comorbidities and smaller tumor size, compared with those who do not undergo such surgery.^47^

Our landmark analysis results suggest that survival duration might affect the association between breast cancer and AD. As the survival period increased, the SHR value approached 1.00, indicating that the risk of AD might differ according to the duration of survival. This finding is consistent with the findings from a Danish study in which cancer survivors showed lower risk of AD in general but AD risk approached that of the general population for those surviving more than 10 years.^5^ In addition, a Swedish study found increased risk of AD among 5-year breast cancer survivors, particularly in older individuals.^6^ These epidemiologic findings are in line with magnetic resonance imaging studies^48,49^ showing decreased brain gray matter density within 1 month after breast cancer chemotherapy that had recovered 1 year later. Short-term follow-up after adjuvant chemotherapy (eg, 1-3 years) revealed no significant differences in regional brain volume compared with that of healthy controls. Meanwhile, another study reported a significant long-term reduction in total brain volume and gray matter volume more than 20 years after postadjuvant chemotherapy for breast cancer.^50^ Based on these findings, we hypothesize that the risk of AD could be lowered shortly after cancer treatment but may equalize as the survival period increases. However, longer landmark periods might reflect selection bias because patients who have survived without AD for extended durations were included, favoring healthier individuals, such as those with early-stage breast cancer, younger age, and fewer comorbidities. Moreover, we could not evaluate the long-term increase in AD risk because our follow-up period was relatively short (maximum, 11 years). Additional studies with long-term observation periods are warranted to examine long-term associations between AD risk and breast cancer survival duration.

The risk of AD is a crucial aspect of overall well-being among breast cancer survivors. Concerns about chemobrain and the long-term adverse effects of breast cancer treatment on cognition are common, but our findings suggest that this treatment does not directly lead to AD. Appropriate management of modifiable risk factors for AD, such as smoking and diabetes, along with standard cancer treatment is a feasible and effective option to lower AD risk among breast cancer survivors. Understanding the potential protective association of breast cancer on AD can enhance surveillance strategies for AD among these survivors.

### Strengths and Limitations

Our study has methodologic strengths over previous studies, including (1) a large study population that enabled us to investigate the risk of AD among breast cancer survivors through various subgroup analyses; (2) consideration of various traditional risk factors of AD, such as smoking, and competing risk; and (3) landmark analyses. However, our study also has limitations to consider. First, the administrative data used in this study do not include detailed clinical information on breast cancer stage or radiation dose and fraction. Second, the number of AD cases could have been underestimated based on the use of *ICD-10* codes. However, considering that patients with breast cancer likely had more frequent medical visits than did controls, underdiagnosis of AD would have been more common in the control group and would have underestimated the actual beneficial association of breast cancer with AD. Third, although we focused on the risk of AD among survivors with operable breast cancer, the inclusion criteria for the study population may have introduced selection bias. Consequently, we may not have captured the risk of AD among elderly patients, those with critical comorbidities, or patients with advanced-stage breast cancer. Fourth, because our study used an age-matched control group to compare AD risk with breast cancer survivors, we adjusted for possible confounding factors for AD risk; however, residual confounding may still exist. Future studies should consider more comprehensive matching approaches for better balance between groups. Fifth, although AD is a slowly progressive disease, our study had a relatively short follow-up period (maximum follow-up, 11 years).

## Conclusions

In this cohort study, breast cancer survivors had an 8% lower risk of AD compared with age-matched controls without cancer. Breast cancer treatment with radiation was associated with a lower risk of AD. Landmark analyses suggest that a longer survival period might attenuate this association.

## Notes

Supplement 1. **eTable 1. **Previous Studies on the Association Between Breast Cancer and Dementia or Alzheimer's Disease **eTable 2. **Cancer Treatment History Among Breast Cancer Survivors **eFigure 1**. The Association Between Breast Cancer Survivors and Alzheimer's Disease by Age Group Was Analyzed Using Gray’s Test **eFigure 2. **Adjusted Subdistribution Hazard Ratios for Risk of Alzheimer’s Disease in Breast Cancer Survivors Compared to the Non-Cancer Control Group by Age Category

Supplement 2.
Data Sharing Statement
