# Supplementary material for: Alzheimer Disease in Breast Cancer Survivors
Source: JAMA Netw Open. 2025 Jun 20;8(6):e2516468. doi: 10.1001/jamanetworkopen.2025.16468 (PMC12181787; doi:10.1001/jamanetworkopen.2025.16468)
Supplement: Supplement 1. — eTable 1. Previous Studies on the Association Between Breast Cancer and Dementia or Alzheimer's Disease eTable 2. Cancer Treatment History Among Breast Cancer Survivors eFigure 1. The Association Between Breast Cancer Survivors and Alzheimer's Disease by Age Group Was Analyzed Using Gray’s Test eFigure 2. Adjusted Subdistribution Hazard Ratios for Risk of Alzheimer’s Disease in Breast Cancer Survivors Compared to the Non-Cancer Control Group by Age Category [file jamanetwopen-e2516468-s001.pdf]

Supplemental Online Content

Jeong SM, Jung W, Cho H, et al. Alzheimer disease in breast cancer survivors. *JAMA Netw Open*. 2025;8(6):e2516468.
doi:10.1001/jamanetworkopen.2025.16468

eTable 1. Previous Studies on the Association Between Breast Cancer and Dementia or Alzheimer's Disease

eTable 2. Cancer Treatment History Among Breast Cancer Survivors

eFigure 1. The Association Between Breast Cancer Survivors and Alzheimer's Disease by Age Group Was Analyzed Using Gray's Test

eFigure 2. Adjusted Subdistribution Hazard Ratios for Risk of Alzheimer's Disease in Breast Cancer Survivors Compared to the Non-Cancer Control Group by Age Category

This supplemental material has been provided by the authors to give readers additional information about their work.

© 2025 Jeong SM et al. *JAMA Network Open*.

eTable 1. Previous Studies on the Association Between Breast Cancer and Dementia or Alzheimer's Disease

Study	Population	outcomes	Follow-up duration	Factor adjusted	considering of competing risk	Key Findings
Wennberg et al., 2023 [6]	26,741 5-year breast cancer survivors aged ≥ 50 (1991-2005) vs. cancer free group	dementia/AD/VaD	30 years	age, education, country of origin	Yes	- No significant association between breast cancer survivorship and risk of all-cause dementia, AD, or VaD.<br><br>- Women diagnosed with cancer after age 65 years had a higher risk of all-cause dementia (SHR, 1.30, 95% CI 1.07-1.58), AD (SHR = 1.35, 95% CI 1.05-1.75), and VaD (SHR = 1.64, 95% CI 1.11-2.43)
Sun et al., 2015 [7]	24,197 breast cancer patients aged ≥ 50 ((2000-2004) vs. cancer free group	dementia	7.4 years	age, comorbidities	Yes	- Patients with breast cancer vs. cancer free group : adjusted HR = 0.95, 95% CI = 0.86–1.04<br><br>- Tamoxifen users vs. not using tamoxifen : adjusted HR = 0.83, 95% CI = 0.69–0.98
Oh et al., 2023 [8]	90,396 breast cancer patients aged ≥ 50 (2009-2010) vs. cataract patients	dementia	104.1±24.0 months	matching for comorbidities and age	No	Breast cancer was a predictor of a lower risk of for dementia (HR, 0.091; 95% CI, 0.075-0.111).
Zhang et al., 2022 [23]	Meta-analysis for all cancers including breast cancer	dementia/AD	NA	age, APOE, education,	NA	- A lower risk of AD in breast cancer (RR:0.93 [0.87–0.99])

© 2025 Jeong SM et al. *JAMA Network Open*.

				smoking for all cancers		- The risk for all dementia with chemotherapy for breast cancer (RR:0.83 [0.73–0.95]).

NA, non-applicable; AD, Alzheimer's disease; VaD, vascular dementia; HR, hazard ratio; CI, confidence interval; RR, relative risk; SHR, subdistribution hazard ratio

© 2025 Jeong SM et al. *JAMA Network Open*.

eTable 2. Cancer Treatment History Among Breast Cancer Survivors

Cancer treatment	Breast cancer survivors, No. (%)<br>70,701
**Chemotherapy**	
Anthracycline	35441 (50.1)
Cyclophosphamide	40144 (56.8)
Fluorouracil	10162 (14.4)
Taxane	18889 (26.7)
Methotrexate or cisplatin	5148 (7.3)
**Trastuzumab**	10241 (14.5)
**Endocrine therapy**	
Tamoxifen	33232 (47.0)
Aromatase inhibitors	21240 (30.0)
**Radiation therapy**	50681 (71.7)

© 2025 Jeong SM et al. *JAMA Network Open*.

eFigure 1. The Association Between Breast Cancer Survivors and Alzheimer's Disease by Age Group Was Analyzed Using Gray's Test

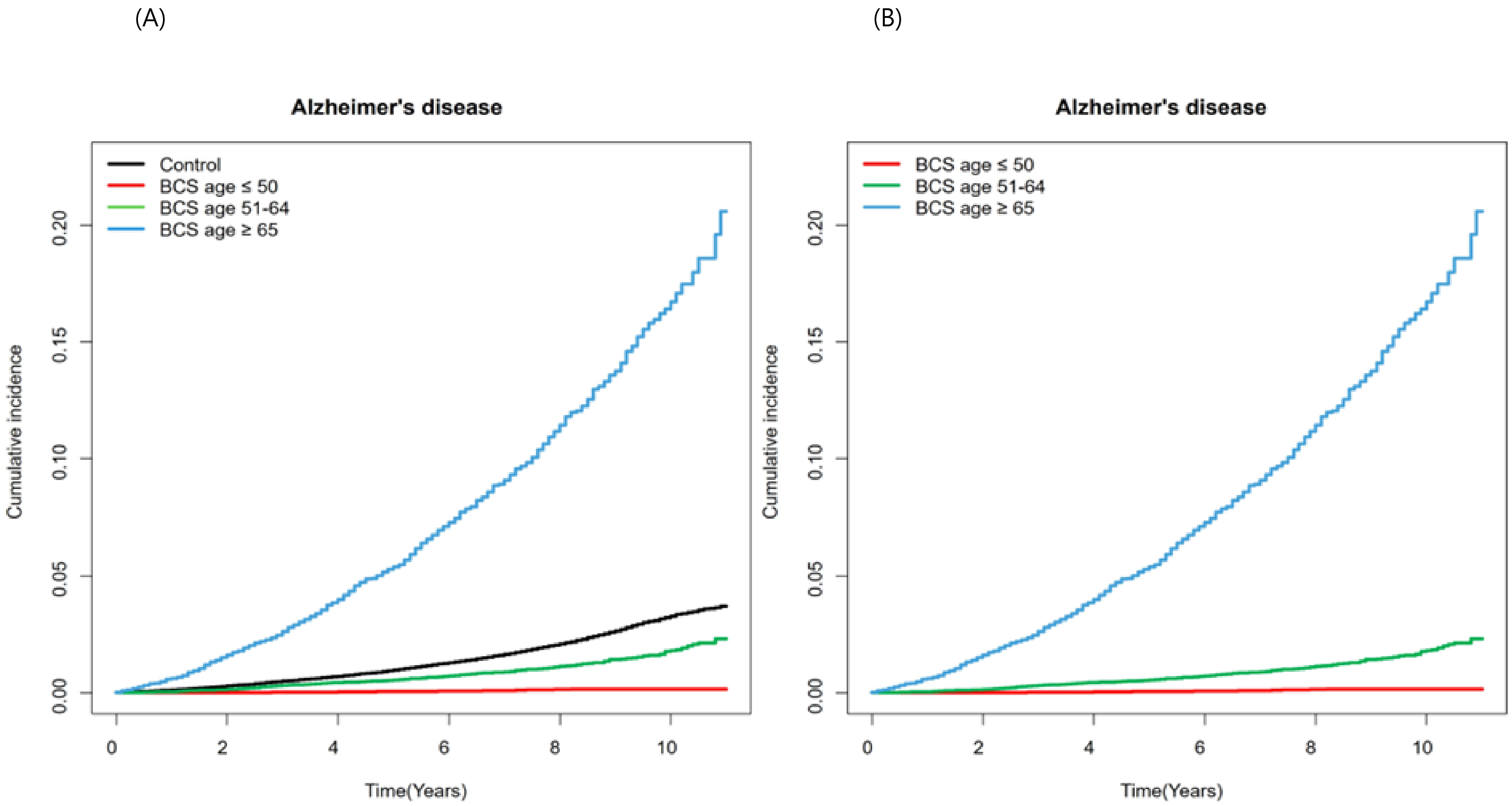

(A) Comparison with the control group and (B) comparison among breast cancer survivors by age group. BCS, breast cancer survivors.

© 2025 Jeong SM et al. *JAMA Network Open*.

eFigure 2. Adjusted Subdistribution Hazard Ratios for Risk of Alzheimer's Disease in Breast Cancer Survivors Compared to the Non-Cancer Control Group by Age Category

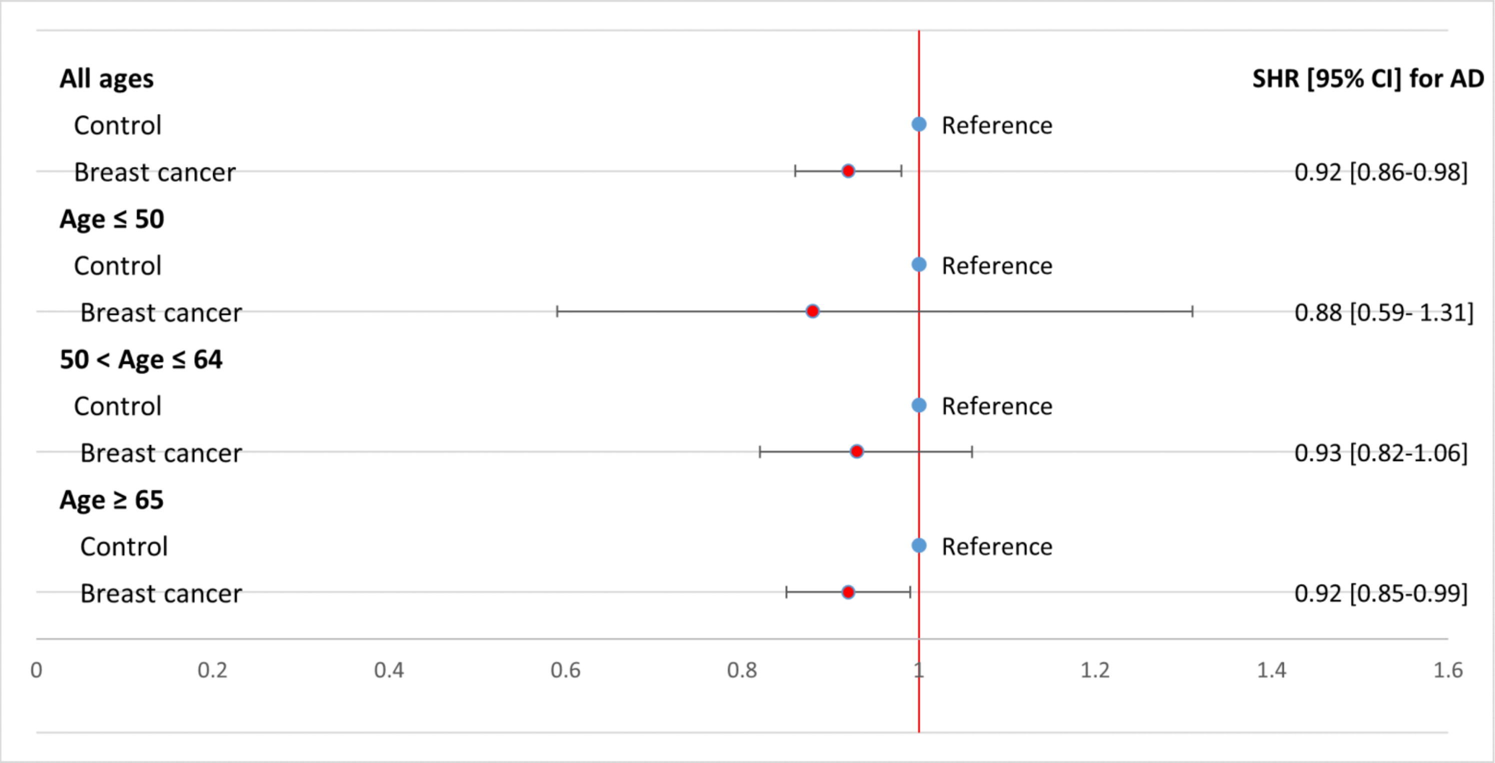

SHR, subdistribution hazard ratio; CI, confidence interval; AD, Alzheimer's disease

© 2025 Jeong SM et al. *JAMA Network Open.*
