# Supplementary material for: Alzheimer Disease in Breast Cancer Survivors
Source: JAMA Netw Open. 2025 Jun 20;8(6):e2516468. doi: 10.1001/jamanetworkopen.2025.16468 (PMC12181787; doi:10.1001/jamanetworkopen.2025.16468)
Supplement: Supplement 2. — Data Sharing Statement [file jamanetwopen-e2516468-s002.pdf]

Data Sharing Statement

Jeong. Alzheimer Disease in Breast Cancer Survivors. *JAMA Netw Open*. Published June 20, 2025. doi:10.1001/jamanetworkopen.2025.16468

Data
Data available: No
